# H_∞_ Optimization of Three-Element-Type Dynamic Vibration Absorber with Inerter and Negative Stiffness Based on the Particle Swarm Algorithm

**DOI:** 10.3390/e25071048

**Published:** 2023-07-12

**Authors:** Ting Gao, Jing Li, Shaotao Zhu, Xiaodong Yang, Hongzhen Zhao

**Affiliations:** 1 Interdisciplinary Research Institute, Faculty of Science, Beijing University of Technology, Beijing 100124, China; gting@emails.bjut.edu.cn (T.G.); zhaohongzhen@emails.bjut.edu.cn (H.Z.); 2 Faculty of Information Technology, Beijing University of Technology, Beijing 100124, China; 3 Faculty of Materials and Manufacturing, Beijing University of Technology, Beijing 100124, China; jxdyang@163.com

**Keywords:** three-element-type DVA, inerter–mass, negative stiffness, H_∞_ optimization, particle swarm optimization algorithm

## Abstract

Dynamic vibration absorbers (DVAs) are extensively used in the prevention of building and bridge vibrations, as well as in vehicle suspension and other fields, due to their excellent damping performance. The reliable optimization of DVA parameters is key to improve their performance. In this paper, an H${}_{\infty }$ optimization problem of a novel three-element-type DVA model including an inerter device and a grounded negative stiffness spring is studied by combining a traditional theory and an intelligent algorithm. Firstly, to ensure the system’s stability, the specific analytical expressions of the optimal tuning frequency ratio, stiffness ratio, and approximate damping ratio with regard to the mass ratio and inerter–mass ratio are determined through fixed-point theory, which provides an iterative range for algorithm optimization. Secondly, the particle swarm optimization (PSO) algorithm is used to further optimize the four parameters of DVA simultaneously. The effects of the traditional fixed-point theory and the intelligent PSO algorithm are comprehensively compared and analyzed. The results verify that the effect of the coupling of the traditional theory and the intelligent algorithm is better than that of fixed-point theory alone and can make the two resonance peaks on the amplitude–frequency response curves almost equal, which is difficult to achieve using fixed-point theory alone. Finally, we compare the proposed model with other DVA models under harmonic and random excitation. By comparing the amplitude–frequency curves, stroke lengths, mean square responses, time history diagrams, variances and decrease ratios, it is clear that the established DVA has a good vibration absorption effect. The research results provide theoretical and algorithm support for designing more effective DVA models of the same type in engineering applications.

## 1. Introduction

Destructive vibrations often lead to problems such as reduced machine performance, reduced reliability, and noise pollution. Dynamic vibration absorbers (DVAs), also known as tuned mass dampers (TMDs), are vibration reduction structures widely used in aerospace, the automotive industry, instrumentation, the construction industry, and construction machinery. In 1909, the first undamped DVA was proposed by Frahm [1]. Due to its narrow band characteristics, its suppression effect on the dynamic response of the primary vibration structure was limited. On this basis, Voigt-type DVA [2], three-element-type DVAs [3,4], and Ren-type DVA [5] were proposed by configuring different linear elements (such as damping elements and stiffness elements), and their vibration reduction performance were further improved. Subsequently, some scholars carried out a series of type improvements on the DVAs.

There are a few previous studies on three-element-type DVAs. Asami et al. [3,4] proposed a three-element-type DVA with Maxwell connections in which the damping in the Voigt-type DVA was replaced by a viscoelastic material; that is, they added a spring in series with the damping element. After using the H${}_{\infty }$ optimization and H${}_{2}$ optimization techniques, it was found that the efficiency of this DVA was superior to Voigt-type DVAs. Nishihara et al. [6] used the Newton–Raphson algorithm to optimize the parameters so that the maximum amplitude amplification coefficient of the linear primary system of the three-element-type DVA was the smallest under a given mass ratio. Song et al. [7] discussed the parameter optimization methods of Voigt-type DVA and three-element-type DVA models based on the H${}_{\infty }$ optimization criterion and compared the performance of these different methods. Chen et al. [8] applied a three-element-type DVA to suppress vibrations in vehicle bodies, and a formula for the design of the suspension parameters of the frame equipment was obtained. In addition, Baduidana et al. [9] investigated a three-element-type DVA with a Kelvin-type connection and used extended fixed-point theory to obtain formulas for the optimized parameters.

With the further development of research on vibration control systems, scholars found that the introduction of negative stiffness springs and inerter components in DVAs can significantly reduce the resonant response, and the effective vibration suppression frequency band can thus be made wider. Some traditional structures to achieve negative stiffness mainly include frame structures, press bar structures, inverted pendulum structures and so on. Some scholars studied the vibration isolation performance of negative stiffness springs and performed a stability analysis on these components [10,11]. They observed that negative stiffness springs were unstable and must be used in combination with positive stiffness springs to effectively exert a vibration isolation effect. Shen et al. [12,13] investigated the optimal design of a Maxwell-type DVA with a grounded negative stiffness spring and proved its effectiveness in performance by conducting a comparison analysis. Negative stiffness components are used in practical engineering applications, such as reducing the vibrations of large mechanical equipment in ships [14], cable vibration control in cable-stayed bridges [15], and seismic protection [16]. Zhang and Xu [17] designed a nonlinear control target and explored a new optimization method for nonlinear aeroelastic systems. In addition, some scholars [18,19] investigated a variety of seismic isolation techniques and applied them to the transformation of actual buildings.

An inerter, also known as an inertial energy storage device or an inertial mass energy storage device, has two independent and free endpoints, and the generated force has a linear relationship with the relative acceleration between the nodes. Compared with traditional damping systems, inerter-based systems are advantageous because they can realize the flexible adjustment of inertia and the adjustment of frequency and they do not change the physical quality of the structure while changing the inertia of the structure. Related research [20] showed that the position of the inerter’s connection had effects on the vibration absorption of the damper. Many scholars proposed a variety of DVAs with inerters and negative stiffness components; they investigated the H${}_{2}$ optimal control and performed stability analyses from the perspective of the seismic response control effect [21,22,23]. Furthermore, some studies also focused on the combined use of inerters and grounded stiffness. Changing the arrangement of control elements produced the generation of a variety of different types of inerter-based systems, such as high-performance three-element-type DVAs [9], inerter-type nonlinear energy sinks [24], and different types of amplified inerter mechanisms [25,26]. As simple amplification mechanisms, levers were often used to amplify the displacement and force in the vibration control system and enhance the system’s performance. Some scholars introduced levers into the inerter absorber to further optimize the structural design and vibration absorption performance of the absorber [27,28]. Liu et al. [29] introduced linear and nonlinear inerters into locally resonant acoustic metamaterials and found that the structure based on the nonlinear inerter was insensitive to changes in the inerter’s coefficient.

With the development of computer and intelligent algorithms, intelligent particle swarm optimization (PSO) algorithms [30], genetic algorithms [31], and other soft computing technologies [32] have been used in theory and practice. The PSO algorithm is a modern optimization method based on swarm intelligence and inspired by research results on artificial life. This algorithm was proposed by Eberhart and Kennedy in 1995 and simulates PSO technology as a form of random optimization under the influence of the social behavior of birds [30]. The optimization problem was regarded as a search problem in a multidimensional space, and each solution was regarded as a particle in the space. Each particle had an initial position and initial velocity. In the search process, each particle constantly adjusted its speed and position and was affected by its own historical optimal solution and group historical optimal solution in order to find the global optimal solution [33,34]. The PSO algorithm can obtain more effective solutions when solving the problem of the multi-parameter optimization of vibration reduction structures. Some researchers [7,35] applied the PSO algorithm to study the parameter optimization of different types of DVAs to obtain an acceptable vibration performance.

Regarding the model design of DVAs, it can be found through a literature review that there are a few previous studies on three-element-type DVAs. More importantly, the performance benefits of the optimal three-element-type DVAs with both inerters and negative stiffness have not been fully studied. For the parameter optimization of DVAs, most scholars directly used fixed-point theory, which failed to achieve equal heights in the resonance peaks of amplitude–frequency curves. Motivated by this, we adopt a more accurate, simpler, and more effective method. The traditional fixed-point theory and the intelligent PSO algorithm are combined to solve the H${}_{\infty }$ optimization problem of a novel three-element-type DVA model. The system parameters are automatically adjusted. The effects of the algorithm before and after optimization are compared, and the effectiveness of the combination of the two methods is verified. This is the innovation of this paper.

This paper is arranged as follows. In Section 2, the established model and optimal parameters are presented. In Section 3, the PSO algorithm is presented, and the results before and after algorithm optimization are analyzed. The influence of the mass ratio and the inerter–mass ratio on the response characteristics is revealed. In Section 4, the vibration reduction performance is compared with other classical DVA models under different levels of excitation. In Section 5, we give our conclusions and discuss future prospects.

## 2. Dynamic Model and Optimum Parameter Expressions

**Figure 1:**
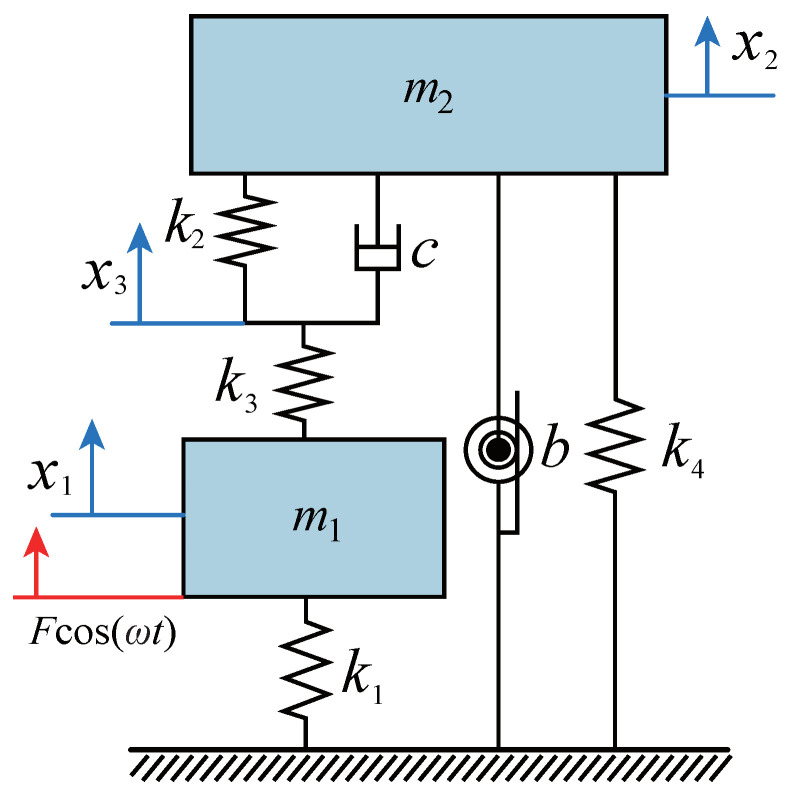
The three-element-type DVA model with inerter and grounded negative stiffness.

Viscoelastic materials are widely used for vibration control in engineering structures. The spring–mass–damper models commonly used in engineering to reflect the viscoelasticity of the system mainly include the Maxwell model and Kelvin model, which are essentially the series or parallel connections of springs and dampers. The three-element layout of springs and dampers proposed in this paper is a typical Kelvin model. In Figure 1, we design a three-element-type inerter-based DVA model with a grounded negative stiffness spring. $m_{1}$ and $k_{1}$ represent the mass and stiffness coefficients of the primary system, respectively. *b* represents the inerter coefficient of the DVA. The so-called three-element-type DVA concept studied in this paper refers to a spring with a stiffness coefficient of $k_{2}$ connected in parallel with a damper (where the damping coefficient is *c*). The upper end of the parallel structure supports the DVA (mass is $m_{2}$), and the lower end connects in series with a spring (where the stiffness coefficient is $k_{3}$). The stiffness coefficient of the negative stiffness spring connecting the ground or infrastructure is $k_{4}$. The displacements of the primary system, DVA, three-element spring, and damping split point are expressed by $x_{1}$, $x_{2}$, and $x_{3}$, respectively.

When the primary system is excited by a harmonic force with amplitude *F* and frequency ω, the governing equation is described as

(1) $$\begin{array}{cc} \; & m_{1}{\ddot{x}}_{1}+k_{1}x_{1}+k_{3}\left(x_{1}-x_{3}\right)=F\cos\left(\omega t\right)\\ \; & m_{2}{\ddot{x}}_{2}+b{\ddot{x}}_{2}+c\left({\dot{x}}_{2}-{\dot{x}}_{3}\right)+k_{2}\left(x_{2}-x_{3}\right)+k_{4}x_{2}=0\\ \; & c\left({\dot{x}}_{3}-{\dot{x}}_{2}\right)+k_{2}\left(x_{3}-x_{2}\right)+k_{3}\left(x_{3}-x_{1}\right)=0 \end{array}$$

### 2.1. The Analytical Solution

We use the following parameter transformations

$$\begin{array}{cc} \; & \omega_{1}=\sqrt{\frac{k_{1}}{m_{1}}},\;\omega_{2}=\sqrt{\frac{k_{2}}{m_{2}}},\;\xi =\frac{c}{2m_{1}\omega_{1}},\;\mu =\frac{m_{2}}{m_{1}}\\ \; & \alpha_{1}=\frac{k_{3}}{k_{1}},\;\alpha_{2}=\frac{k_{4}}{k_{1}},\;f=\frac{F}{m_{1}},\;\beta =\frac{b}{m_{1}} \end{array}$$

where $\omega_{1}$ and $\omega_{2}$ represent the natural frequencies of the primary system and the absorber system, respectively. ξ represents the damping ratio of the DVA. μ represents the mass ratio. $\alpha_{1}$ and $\alpha_{2}$ represent the corresponding ratios of the spring constants. *f* represents the amplitude-to-mass ratio. β represents the inerter-to-mass ratio.

Equation (1) can be rewritten as

(2) $$\begin{array}{cc} \; & {\ddot{x}}_{1}+\omega_{1}^{2}x_{1}+\alpha_{1}\omega_{1}^{2}\left(x_{1}-x_{3}\right)=f\cos\left(\omega t\right)\\ \; & \mu {\ddot{x}}_{2}+\beta {\ddot{x}}_{2}+2\omega_{1}\xi \left({\dot{x}}_{2}-{\dot{x}}_{3}\right)+\mu \omega_{2}^{2}\left(x_{2}-x_{3}\right)+\alpha_{2}\omega_{1}^{2}x_{2}=0\\ \; & 2\omega_{1}\xi \left({\dot{x}}_{3}-{\dot{x}}_{2}\right)+\mu \omega_{2}^{2}\left(x_{3}-x_{2}\right)+\alpha_{1}\omega_{1}^{2}\left(x_{3}-x_{1}\right)=0 \end{array}$$

Assuming that steady-state solutions are in the form of

(3) $$\begin{array}{c} x_{i}=X_{i}e^{j\omega t},\;i=1,2,3, \end{array}$$

then one can obtain the following by substituting them into Equation (2):

(4) $$\begin{array}{c} X_{1}=\frac{f\left(b_{2}c_{3}-b_{3}c_{2}\right)}{\det\left\{\sum_{i=1}^{3}\left[\partial_{1,\;i}^{3,\;3}\left(a_{i}\right)+\partial_{2,\;i}^{3,\;3}\left(b_{i}\right)+\partial_{3,\;i}^{3,\;3}\left(c_{i}\right)\right]\right\}} \end{array}$$

$\partial_{p\;,\;q}^{m\;,\;n}\left(J\right)$ is an m×n matrix in which the (p,q)-th block is J and the other blocks are zero matrices [36]. By a simple calculation, we have

(5) $$\begin{array}{c} \begin{array}{cc} a_{1} & =-\omega^{2}+\left(\alpha_{1}+1\right)\omega_{1}^{2},\;a_{2}=b_{1}=0,\;a_{3}=c_{1}=-\alpha_{1}\omega_{1}^{2}\\ b_{2} & =-\mu \omega^{2}-\beta \omega^{2}+2\omega_{1}\xi j\omega +\mu \omega_{2}^{2}+\alpha_{2}\omega_{1}^{2}\\ b_{3} & =c_{2}=-2\omega_{1}\xi j\omega -\mu \omega_{2}^{2},\;c_{3}=2\omega_{1}\xi j\omega +\mu \omega_{2}^{2}+\alpha_{1}\omega_{1}^{2} \end{array} \end{array}$$

We then insert the following parameters,

$$\begin{array}{c} \lambda =\frac{\omega }{\omega_{1}},\;\nu =\frac{\omega_{2}}{\omega_{1}},\;X_{st}=\frac{F}{k_{1}}, \end{array}$$

where λ and ν represent the forced frequency ratio and natural frequency ratio. $X_{st}$ represents the static deformation of the primary system. Let *A* be the amplitude amplification factor of the primary system in dimensionless form; that is,

(6) $$\begin{array}{c} A^{2}=|\frac{X_{1}}{X_{st}}{|}^{2}=\frac{d_{1}^{2}+d_{2}^{2}\xi^{2}}{d_{3}^{2}+d_{4}^{2}\xi^{2}} \end{array}$$

where

(7) $$\begin{array}{cc} d_{1}= & -\lambda^{2}\left(\alpha_{1}+\mu \nu^{2}\right)\gamma +\left(\alpha_{1}+\alpha_{2}\right)\mu \nu^{2}+\alpha_{1}\alpha_{2}\\ d_{2}= & -2\lambda (\gamma \lambda^{2}-\alpha_{1}-\alpha_{2})\\ d_{3}= & \left(\alpha_{1}+\mu \nu^{2}\right)\gamma \lambda^{4}+h_{1}\mu \nu^{2}+\alpha_{1}\alpha_{2}\\ \; & -\left[\left(\alpha_{1}+1\right)\mu^{2}\nu^{2}+h_{2}\mu \nu^{2}+\alpha_{1}\alpha_{2}+\alpha_{1}\beta +\alpha_{1}\mu \right]\lambda^{2}\\ d_{4}= & 2\lambda \left\{\gamma \lambda^{4}-\left[\left(\alpha_{1}+1\right)\gamma +\alpha_{1}+\alpha_{2}\right]\lambda^{2}+h_{1}\right\}\\ h_{1}= & \alpha_{1}\alpha_{2}+\alpha_{1}+\alpha_{2},\;h_{2}=\alpha_{1}\beta +\alpha_{1}+\alpha_{2}+\beta ,\;\gamma =\beta +\mu \end{array}$$

### 2.2. Closed-Form Solutions to $\nu_{opt}$ and $\alpha_{1opt}$

It is clear from Equation (6) that the amplitude amplification factor of the primary system contains the following six variables: μ, β, $\alpha_{1}$, $\alpha_{2}$, ν, and ξ. Among them, μ and β are the parameters of the system itself and generally do not need to be optimized. The remaining four parameters can be optimized according to different optimization methods to reduce the vibration of the primary system. The ultimate goal of H${}_{\infty }$ optimization is to minimize the maximum amplitude amplification factor *A* of the primary system under harmonic excitation.

**Figure 2:**
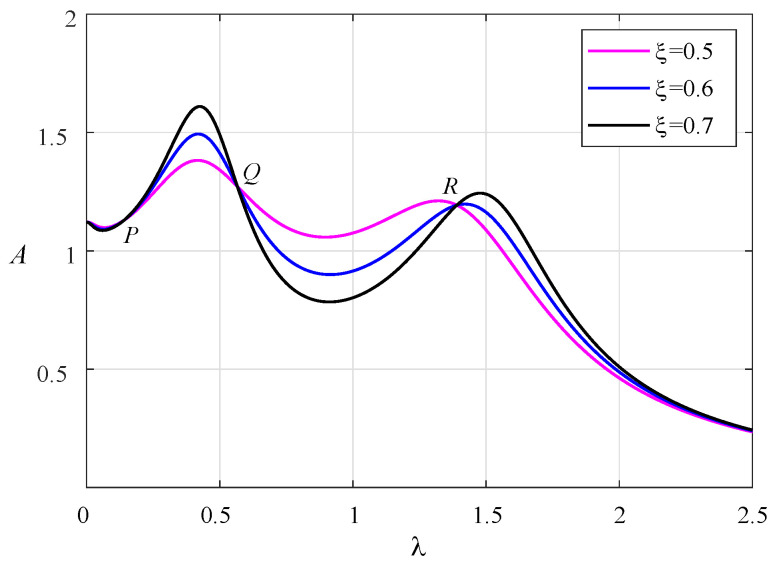
The normalized amplitude–frequency curves can be found under different damping ratios with μ=0.1, β=1.8, ν=1, $\alpha_{1}=1.2$, $\alpha_{2}=-0.05$.

According to Equation (6), the corresponding amplitude–frequency response curves are drawn in Figure 2. The damping ratios are adopted as 0.5, 0.6, and 0.7. There exist three fixed points, *P*, *Q*, and *R*, on all the curves, which are independent of the damping ratio. Based on fixed-point theory, we can obtain the following relationship from Equation (6):

(8) $$\begin{array}{c} \left|\frac{d_{1}}{d_{3}}|=|\frac{d_{2}}{d_{4}}\right| \end{array}$$

Substituting variables into Equation (8), we obtain

(9) $$\begin{array}{c} q_{1}\lambda^{6}+q_{2}\lambda^{4}+q_{3}\lambda^{2}+q_{4}=0 \end{array}$$

where

(10) $$\begin{array}{c} \begin{array}{cc} q_{1}= & -2\left[\mu^{3}\nu^{2}+\left(2\beta \nu^{2}+\alpha_{1}\right)\mu^{2}+\left(\beta^{2}\nu^{2}+2\alpha_{1}\beta \right)\mu +\alpha_{1}\beta^{2}\right]\\ q_{2}= & 2\mu^{3}\nu^{2}\left(\alpha_{1}+1\right)+\left(4\nu^{2}h_{2}+\alpha_{1}^{2}+2\alpha_{1}\right)\mu^{2}\\ \; & +\left\{2\nu^{2}\left[\left(\alpha_{1}+1\right)\beta^{2}+2\left(\alpha_{1}+\alpha_{2}\right)\beta \right]+2\alpha_{1}^{2}\left(\beta +1\right)+4\alpha_{1}\left(\alpha_{2}+\beta \right)\right\}\mu \\ \; & +\left(\alpha_{1}^{2}+2\alpha_{1}\right)\beta^{2}+2\alpha_{1}\left(\alpha_{1}+2\alpha_{2}\right)\beta \\ q_{3}= & -2\left\{h_{3}\mu^{2}\nu^{2}+\left[h_{3}\beta +{\left(\alpha_{1}+\alpha_{2}\right)}^{2}\right]\mu \nu^{2}+h_{4}\gamma +\alpha_{1}\alpha_{2}\left(\alpha_{1}+\alpha_{2}\right)\right\}\\ q_{4}= & 2\left[\left(1+\alpha_{2}\right)\alpha_{1}^{2}+\left(\alpha_{2}^{2}+2\alpha_{2}\right)\alpha_{1}+\alpha_{2}^{2}\right]\mu \nu^{2}+\left(\alpha_{2}^{2}+2\alpha_{2}\right)\alpha_{1}^{2}+2\alpha_{1}\alpha_{2}^{2}\\ h_{3}= & \alpha_{1}^{2}+2\alpha_{1}\alpha_{2}+2\alpha_{1}+2\alpha_{2},\;h_{4}=\alpha_{1}^{2}\alpha_{2}+\alpha_{1}^{2}+2\alpha_{1}\alpha_{2} \end{array} \end{array}$$

When ξ=0, we can obtain

(11) $$\begin{array}{c} A=|\frac{X_{1}}{X_{st}}|=|\frac{-\left(\alpha_{1}+\mu \nu^{2}\right)\gamma \lambda^{2}+\left(\alpha_{1}+\alpha_{2}\right)\mu \nu^{2}+\alpha_{1}\alpha_{2}}{-\left(\alpha_{1}+\mu \nu^{2}\right)\gamma \lambda^{4}+h_{5}\lambda^{2}-h_{1}\mu \nu^{2}-\alpha_{1}\alpha_{2}}| \end{array}$$

where

$$\begin{array}{c} h_{5}=\left(\alpha_{1}+1\right)\mu^{2}\nu^{2}+h_{2}\mu \nu^{2}+\alpha_{1}\left(\gamma +\alpha_{2}\right) \end{array}$$

When ξ→∞, we can obtain

(12) $$\begin{array}{c} A=|\frac{X_{1}}{X_{st}}|=|\frac{\gamma \lambda^{2}-\alpha_{1}-\alpha_{2}}{\gamma \lambda^{4}-\left[\gamma \left(\alpha_{1}+1\right)+\alpha_{1}+\alpha_{2}\right]\lambda^{2}+h_{1}}| \end{array}$$

In order to facilitate the addition and subtraction of the two equal fractions represented by Equations (11) and (12), we multiply the numerator and denominator of Equation (12) by $\left(\alpha_{1}+\mu \nu^{2}\right)$ at the same time so as to eliminate $\lambda^{4}$ from the denominator. We thus obtain

(13) $$\begin{array}{c} A=|\frac{X_{1}}{X_{st}}|=|\frac{\left(\alpha_{1}+\mu \nu^{2}\right)\left(\gamma \lambda^{2}-\alpha_{1}-\alpha_{2}\right)}{\left(\alpha_{1}+\mu \nu^{2}\right)\left\{\gamma \lambda^{4}-\left[\gamma \left(\alpha_{1}+1\right)+\alpha_{1}+\alpha_{2}\right]\lambda^{2}+h_{1}\right\}}| \end{array}$$

Hence, by adding Equations (11) and (13), we have

$$\begin{array}{c} A=|\frac{X_{1}}{X_{st}}|=|\frac{-2\gamma \left(\alpha_{1}+\mu \nu^{2}\right)\lambda^{2}+2\mu \nu^{2}\left(\alpha_{1}+\alpha_{2}\right)+\alpha_{1}\left(\alpha_{1}+2\alpha_{2}\right)}{\alpha_{1}^{2}\left[\left(-\gamma -1\right)\lambda^{2}+\alpha_{2}+1\right]}| \end{array}$$

Assuming that $\lambda_{P}^{2}$, $\lambda_{Q}^{2}$, and $\lambda_{R}^{2}$ are three roots of Equation (9), the vertical ordinates of the three fixed points are expressed as

(14) $$\begin{array}{c} \begin{array}{cc} |\frac{X_{1}}{X_{st}}{|}_{P} & =|\frac{-2\gamma \left(\alpha_{1}+\mu \nu^{2}\right)\lambda_{P}^{2}+2\mu \nu^{2}\left(\alpha_{1}+\alpha_{2}\right)+\alpha_{1}\left(\alpha_{1}+2\alpha_{2}\right)}{\alpha_{1}^{2}\left[\left(-\gamma -1\right)\lambda_{P}^{2}+\alpha_{2}+1\right]}|\\ |\frac{X_{1}}{X_{st}}{|}_{Q} & =|-\frac{-2\gamma \left(\alpha_{1}+\mu \nu^{2}\right)\lambda_{Q}^{2}+2\mu \nu^{2}\left(\alpha_{1}+\alpha_{2}\right)+\alpha_{1}\left(\alpha_{1}+2\alpha_{2}\right)}{\alpha_{1}^{2}\left[\left(-\gamma -1\right)\lambda_{Q}^{2}+\alpha_{2}+1\right]}|\\ |\frac{X_{1}}{X_{st}}{|}_{R} & =|\frac{-2\gamma \left(\alpha_{1}+\mu \nu^{2}\right)\lambda_{R}^{2}+2\mu \nu^{2}\left(\alpha_{1}+\alpha_{2}\right)+\alpha_{1}\left(\alpha_{1}+2\alpha_{2}\right)}{\alpha_{1}^{2}\left[\left(-\gamma -1\right)\lambda_{R}^{2}+\alpha_{2}+1\right]}| \end{array} \end{array}$$

The first step is to adjust the vertical coordinates of *P* and *R* to the same height, i.e.,

(15) $$\begin{array}{c} |\frac{X_{1}}{X_{st}}{|}_{P}=|\frac{X_{1}}{X_{st}}{|}_{R} \end{array}$$

The optimum frequency ratio can be obtained by Equation (15) as

(16) $$\begin{array}{c} \nu =\sqrt{\frac{\alpha_{1}\left[2\left(\alpha_{2}-\gamma \right)+\alpha_{1}\left(\gamma +1\right)\right]}{2\mu (-\alpha_{2}+\gamma -\alpha_{1}\left(\gamma +1\right))}} \end{array}$$

Substituting Equation (16) into Equation (9), the abscissa of the three fixed points is

(17) $$\begin{array}{c} \begin{array}{cc} \lambda_{P}^{2}= & \frac{\gamma -\sqrt{G}}{\gamma }\\ \lambda_{Q}^{2}= & \frac{1+\alpha_{2}}{1+\gamma }\\ \lambda_{R}^{2}= & \frac{\gamma +\sqrt{G}}{\gamma } \end{array} \end{array}$$

where $G=\gamma \left(\alpha_{1}^{2}+\gamma \right)+\left(\alpha_{1}+\alpha_{2}\right)\left(\alpha_{1}+\alpha_{2}-2\gamma \right)$.

Thus, Equation (14) becomes

(18) $$\begin{array}{c} \begin{array}{cc} |\frac{X_{1}}{X_{st}}{|}_{P,R} & =|\frac{\gamma }{\alpha_{1}+\alpha_{2}+\left(\alpha_{1}-1\right)\gamma }|\\ |\frac{X_{1}}{X_{st}}{|}_{Q}\;\;\; & =|\frac{\left[\alpha_{1}+\alpha_{2}+\left(\alpha_{2}-1\right)\gamma \right]\left(\gamma +1\right)}{{\left(\alpha_{2}-\gamma \right)}^{2}}| \end{array} \end{array}$$

The second step is to adjust the ordinate of point *P* (or *R*) and point *Q* to the same height to obtain the optimal stiffness ratio.

$$\begin{array}{c} \begin{array}{cc} \alpha_{1a} & =\frac{\left[\gamma +\sqrt{\gamma (\gamma +1)}+1\right]\left(\gamma -\alpha_{2}\right)}{{\left(\gamma +1\right)}^{2}}\\ \alpha_{1b} & =\frac{\left[\gamma -\sqrt{\gamma (\gamma +1)}+1\right]\left(\gamma -\alpha_{2}\right)}{{\left(\gamma +1\right)}^{2}} \end{array} \end{array}$$

By simple derivation, we find that $\alpha_{1b}<0$ under certain parameters, so $\alpha_{1a}$ is selected as the optimal stiffness ratio.

(19) $$\begin{array}{c} \begin{array}{c} \alpha_{1opt}=\frac{\left[\gamma +\sqrt{\gamma (\gamma +1)}+1\right]\left(\gamma -\alpha_{2}\right)}{{\left(\gamma +1\right)}^{2}} \end{array} \end{array}$$

Then, we substitute $\alpha_{1opt}$ into Equation (16) to obtain

(20) $$\begin{array}{c} \nu_{opt}=\sqrt{\frac{\gamma -\alpha_{2}}{2\mu \sqrt{\gamma {\left(\gamma +1\right)}^{3}}}} \end{array}$$

The ordinate of the response at the three fixed points of *P*, *Q*, and *R* can be obtained

(21) $$\begin{array}{c} |\frac{X_{1}}{X_{st}}{|}_{P,Q,R}^{2}=\frac{\gamma (\gamma +1)}{{\left(\alpha_{2}-\gamma \right)}^{2}} \end{array}$$

### 2.3. Closed-Form Solutions to $\alpha_{2opt}$ and $\xi_{opt}$

The proposed model contains a negative stiffness spring, which exhibits negative stiffness subjected to preload. Improper negative stiffness values will make the system unstable, so the negative stiffness term must be optimized. We can see that when the primary system displacement is equal to the response value at the fixed points due to preloading, the system will be stable; that is,

(22) $$\begin{array}{c} |\frac{X_{1}}{X_{st}}{|}_{\lambda =0}^{2}=|\frac{X_{1}}{X_{st}}{|}_{P,Q,R}^{2} \end{array}$$

where

$$\begin{array}{c} |\frac{X_{1}}{X_{st}}{|}_{\lambda =0}^{2}=\frac{{\left[\mu \nu^{2}\left(\alpha_{1}+\alpha_{2}\right)+\alpha_{1}\alpha_{2}\right]}^{2}}{{\left(\mu \nu^{2}h_{1}+\alpha_{1}\alpha_{2}\right)}^{2}} \end{array}$$

Solving Equation (22), the possible optimal negative stiffness ratios are obtained as

(23) $$\begin{array}{c} \begin{array}{cc} \alpha_{2a} & =\frac{\gamma \left(\gamma +2\right)-\sqrt{M}}{\gamma +2}\\ \alpha_{2b} & =\frac{\gamma \left(\gamma +2\right)+\sqrt{M}}{\gamma +2}\\ \alpha_{2c} & =\frac{\gamma \left[2\sqrt{\gamma (\gamma +1)}-2\gamma -1\right]}{2\sqrt{\gamma (\gamma +1)}-\gamma }\\ \alpha_{2d} & =-1 \end{array} \end{array}$$

where $M=\gamma^{4}+4\gamma^{3}+5\gamma^{2}+2\gamma$.

According to Equation (6), the dimensionless natural frequencies expression of the coupled system with regard to the inerter–mass ratio, β, and the negative stiffness ratio, $\alpha_{2}$, can be obtained as

(24) $$\begin{array}{c} \begin{array}{cc} \; & \Omega_{1,2}=\frac{\sqrt{2}}{2}\frac{\omega_{1}}{\sqrt{\gamma (\alpha_{1}\omega_{1}^{2}+\mu \omega_{2}^{2})}}\sqrt{\Phi_{1}\mp \sqrt{\Phi_{1}^{2}-\Phi_{2}}}\\ \; & \Phi_{1}=\left(\gamma +\alpha_{2}\right)\alpha_{1}\omega_{1}^{2}+\left[\gamma \left(\alpha_{1}+1\right)+\alpha_{1}+\alpha_{2}\right]\mu \omega_{2}^{2}\\ \; & \Phi_{2}=4\gamma \left(\alpha_{1}^{2}\alpha_{2}\omega_{1}^{4}+h_{1}\mu^{2}\omega_{2}^{4}+h_{4}\mu \omega_{1}^{2}\omega_{2}^{2}\right) \end{array} \end{array}$$

Substituting Equation (23) into Equation (24), we can see that $\alpha_{2a}$, $\alpha_{2b}$, and $\alpha_{2d}$ make the natural frequency of the coupled system imaginary. $\alpha_{2b}$ also makes the optimal natural frequency ratio $\nu_{opt}$ an imaginary number. Hence, the optimal grounded negative stiffness ratio is chosen to be $\alpha_{2c}$, such that

(25) $$\begin{array}{c} \alpha_{2opt}=\alpha_{2c}=\frac{\gamma \left[2\sqrt{(\gamma +1)\gamma }-2\gamma -1\right]}{2\sqrt{(\gamma +1)\gamma }-\gamma } \end{array}$$

**Figure 3:**
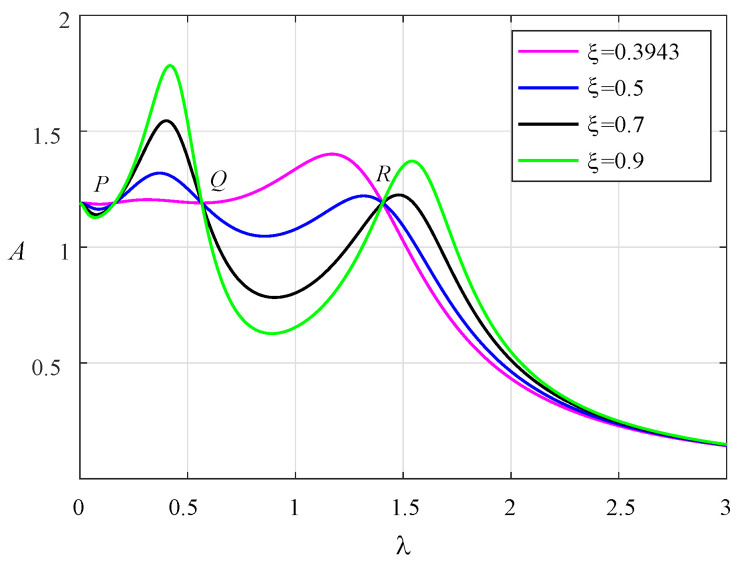
The normalized amplitude–frequency curves of system with μ=0.1, β=1.8, ν=1.2034, $\alpha_{1}=1.2302$, and $\alpha_{2}=-0.0716$.

As shown in Figure 3, the three fixed points have been adjusted to the same height at this time. When the two resonance peaks are adjusted to the same height, the optimal damping ratio can be obtained. When the two resonance peaks are almost at the same height, the fixed point *Q* will be very close to the horizontal point tangent to the amplitude–frequency curve. Therefore, according to the following extremum conditions at the point *Q*, the optimal damping ratio can be deduced.

(26) $$\begin{array}{c} \begin{array}{cc} \; & \frac{\partial A^{2}}{\partial \lambda^{2}}=0\\ \; & \alpha_{1opt}=\frac{\left[\gamma +1+\sqrt{\gamma (\gamma +1)}\right]\left(\gamma -\alpha_{2}\right)}{{\left(\gamma +1\right)}^{2}}\\ \; & \nu_{opt}=\sqrt{\frac{\gamma -\alpha_{2}}{2\mu \sqrt{\gamma {\left(\gamma +1\right)}^{3}}}}\\ \; & \lambda_{Q}^{2}=\frac{\alpha_{2}+1}{\gamma +1} \end{array} \end{array}$$

Thus, the approximate optimal damping ratio can be obtained:

(27) $$\begin{array}{c} \xi_{\mathrm{opt}}≅\sqrt{\frac{\left[e_{1}-g-4\gamma +\sqrt{{\left(\gamma +1\right)}^{2}\left(e_{2}g+e_{3}\right)}\right]{\left(\alpha_{2}-\gamma \right)}^{2}}{16\gamma {\left(\gamma +1\right)}^{2}\left(\gamma +g+1\right)\left(\alpha_{2}+1\right)}} \end{array}$$

where

$$\begin{array}{c} \begin{array}{cc} \; & g=\sqrt{(\gamma +1)\gamma },\;e_{1}=-4\left(2g^{3}+2\gamma^{3}+3\gamma^{2}\right)\\ \; & e_{2}=8\left(16\gamma^{3}+24\gamma^{2}+10\gamma +1\right),\;e_{3}=32\left(4\gamma^{4}+8\gamma^{3}+5\gamma^{2}+\gamma \right)+1 \end{array} \end{array}$$

### 2.4. Three-Dimensional Diagrams of the Optimal Parameters

**Figure 4:**
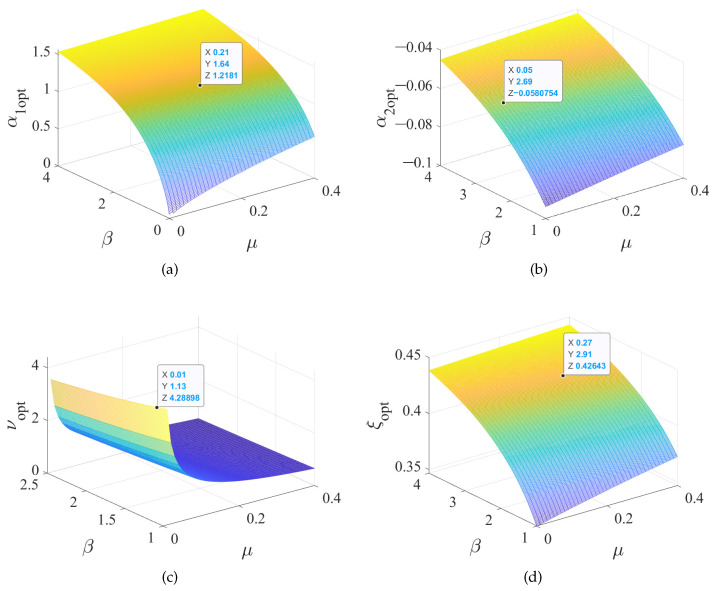
A 3D surface diagram of the relationship between DVA optimal design parameters and system parameters: (**a**) $\alpha_{1opt}$, (**b**) $\alpha_{2opt}$, (**c**) $\nu_{opt}$, and (**d**) $\xi_{opt}$.

The optimal parameter design expressions are given by Equations (19), (20), (25), and (27). We use μ and β as the abscissa and ordinate and draw the three-dimensional diagrams of the four optimal parameters, as shown in Figure 4. When β increases, the value of each design parameter gradually increases. When the mass ratio μ increases, the values of $\alpha_{1}$, $\alpha_{2}$, and ξ increase, while ν decreases.

## 3. Particle Swarm Optimization Algorithm and Comparative Analysis

In the previous section, a set of local approximate optimal parameter formulas of the DVA were calculated through fixed-point theory. An algorithm for particle swarm optimization is employed in this section to iteratively optimize multiple variables within a given scope, thereby ascertaining the most suitable vibration absorption parameters for the DVA.

### 3.1. The Optimal Parameters Obtained by the Particle Swarm Optimization Algorithm

The optimal parameters obtained by fixed-point theory can provide a reference range for parameter regulation for intelligent algorithm regulation. We selected different mass ratios μ and inerter–mass ratios β; then, the local optimal parameter values calculated based on fixed-point theory were used as the reference values of the algorithm optimization variables, as shown in Table 1.

**Figure 5:**
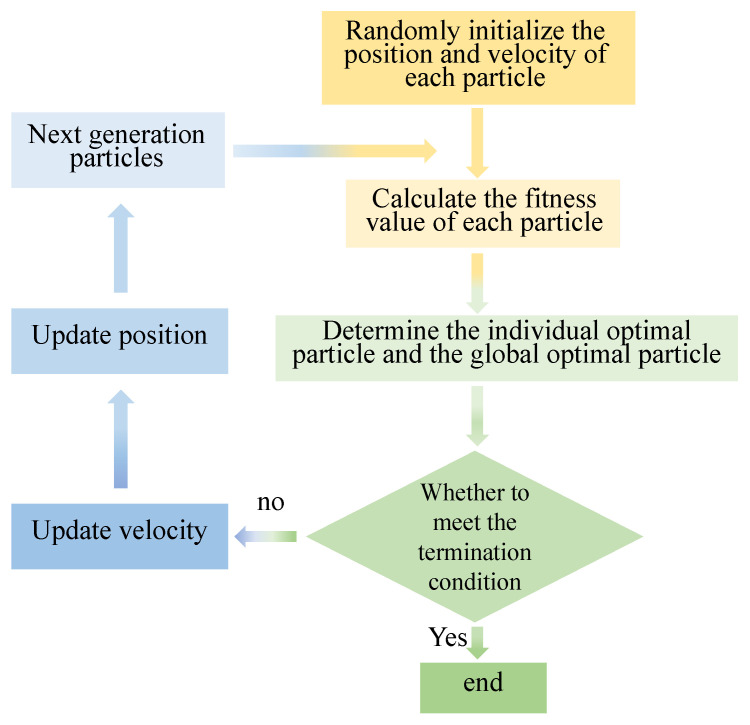
Particle swarm optimization algorithm flow chart.

The PSO algorithm is a search algorithm that relies on collective collaboration. It is an intelligent optimization algorithm. Figure 5 shows the basic idea of the algorithm. An objective function determines the fitness of all particles, and a velocity determines their flight direction and distance. Particles know their current optimal value and current position, and each particle also knows the best position found by all particles in the current population. The particles determine their next action through their own and peer experience, and they find the optimal solution through iteration. The particles will alter their velocity and location to discover the single optimal solution and the global optimal solution. When the iteration count is maximized, the particle swarm can locate the optimal spot and output the ideal parameter value according to the termination conditions of the iteration.

The PSO algorithm was solved by MATLAB programming. The objective function is defined as

(28) $$\begin{array}{c} A_{0}=\min_{(\alpha_{1},\alpha_{2},\nu ,\xi )\in \tau }\left(\;\max_{\lambda \in (0,3)}\;A\left(\alpha_{1},\alpha_{2},\nu ,\xi \right)\right) \end{array}$$

where τ represents the change interval of the DVA’s parameters. We set the particle dimension as 4, corresponding to the parameters $\alpha_{1}$, $\alpha_{2}$, ν, and ξ to be determined. It can be clearly seen from Equation (28) that the optimization objective function in this paper is a specific explicit function, but it needs to optimize four adjustable parameters at the same time. It is usually difficult to obtain analytical results when the traditional fixed-point theory is used to solve the optimal damping ratio. Generally, the approximate value of the damping ratio is given, which leads to the fact that the two resonance peaks cannot achieve the same height on the amplitude–frequency response curves. The PSO algorithm can optimize four variables at the same time so that the resonance peaks of the amplitude–frequency curves are equal, which is difficult to achieve by traditional theoretical methods. This method can also achieve higher accuracy and faster calculation speeds. Based on the advantages of the PSO algorithm in data processing, we further optimize the optimal parameters and realize an automatic parameter adjustment strategy, which is the novelty of this paper in dealing with parameter optimization problems.

We selected the total number of particles as 60, the maximum iteration times as 1000, and the learning factor as $c_{1}=c_{2}=2$. In addition, we set the maximum inertia weight as 0.6 and the minimum inertia weight as 0.4. By investigating the amplitude of the amplitude–frequency response curve, the system will generate two formants according to the parameter values, and it is optimal for the two formants to achieve the same height.

**Figure 6:**
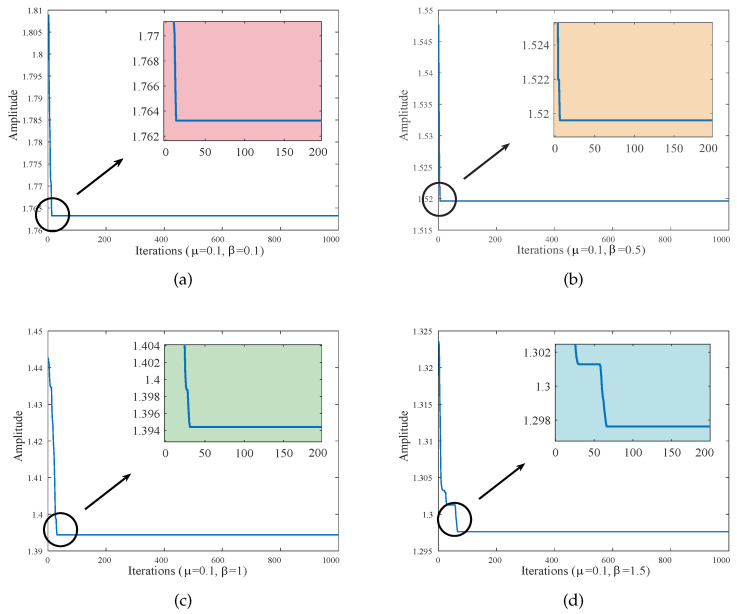
Iterations when optimizing four variables (μ=0.1): (**a**) β= 0.1; (**b**) β= 0.5; (**c**) β= 1; and (**d**) β= 1.5.

Obtaining the maximum amplitude amplification factor *A* of the primary system in the *i*-th iteration is the initial step in the design of an intelligent optimization algorithm for the particle swarm technique. Subsequently, the four optimal parameters that minimize the maximum amplitude in all iterations are output. These are the optimal parameters we seek, which will make the two resonance peaks of the amplitude-frequency response curve reach the same height. We can observe that when μ=0.1 and β=0.1, the optimal parameters of the DVA in the given optimization range are $\alpha_{1}=0.38226$, $\alpha_{2}=-0.11283$, ν=1.60040, and ξ=0.24288. When μ=0.1 and β=0.5, the optimal parameters of the DVA in the given optimization range are $\alpha_{1}=0.71506$, $\alpha_{2}=-0.11568$, ν=1.53110, and ξ=0.36068. Similarly, the optimal parameters obtained by algorithm optimization under different values of μ and β are listed in Table 2. Figure 6 shows the iterative curve of the PSO algorithm for μ=0.1. As is evident from Figure 6, the amplitude diminishes drastically in the initial iteration phase. The amplitude of the primary system eventually flattens out as the number of iterations increases. At the same mass ratio, increasing the inerter–mass ratio can reduce the amplitudes. It should be noted that the initial range of parameters given in the iterative process is very important and is directly related to whether the detected optimal value is accurate.

### 3.2. Effectiveness Analysis of the Particle Swarm Optimization Algorithm

**Figure 7:**
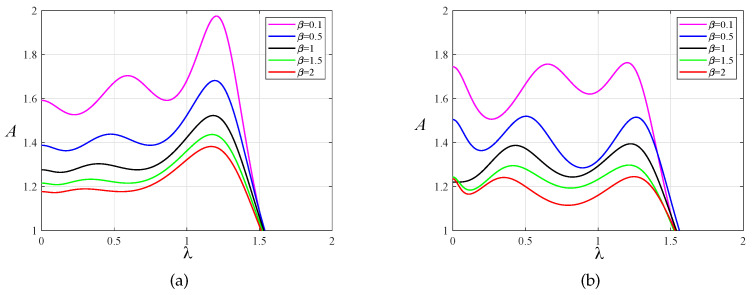
The amplitude–frequency response curves of the primary system when μ=0.1 for the two optimization methods: (**a**) fixed-point theory optimization and (**b**) particle swarm optimization.

**Figure 8:**
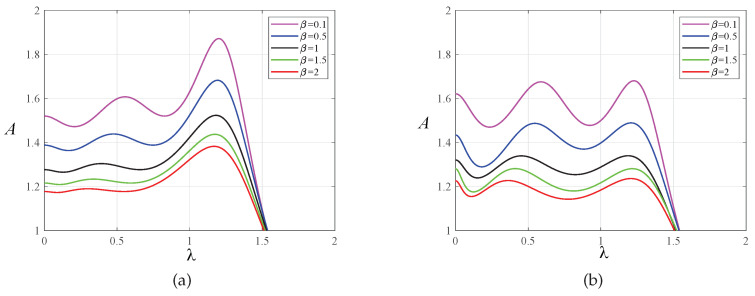
The amplitude–frequency response curves of the primary system when μ=0.2 for the two optimization methods: (**a**) fixed-point theory optimization and (**b**) particle swarm optimization.

In order to prove the effectiveness of the multi-parameter iterative optimization carried out by the PSO algorithm, we compared the amplitude–frequency response curves of the primary system before and after optimization using the PSO algorithm, as shown in Figure 7 and Figure 8. Through this comparison, we found that after optimization using fixed-point theory, the two resonance peaks of the amplitude-frequency curves did not reach the same horizontal height. The PSO algorithm made the two peaks almost equal, and the amplitude was significantly reduced. The change in amplitude between the two resonance peaks was relatively small, and no large fluctuation occurred, indicating good stability.

As shown in Table 3, we compared the specific values of the response characteristics of the primary system in a more comprehensive way, including the maximum value of the amplitude–frequency curve, $A_{max}$; the horizontal coordinates $\lambda_{peak1}$ and $\lambda_{peak2}$, which correspond to the two peaks; the horizontal distance between the two resonance peaks, $|\lambda_{peak2}-\lambda_{peak1}|$; and the mean square response, $\sigma_{opt}^{2}$. Through the comparison of these indicators, the following detailed conclusions can be drawn:

(1) Under the same mass ratio μ and inerter–mass ratio β, the $A_{max}$ obtained with the PSO algorithm is significantly lower than that obtained with the optimization method based on fixed-point theory, and the mean square response value $\sigma_{opt}^{2}$ is also low. That is, the vibration state of the system under external excitation after algorithm optimization is more stable than before algorithm optimization. However, in the case of μ and β taking some parameters, the distance between the two resonance peaks becomes shorter after intelligent algorithm optimization. In practical engineering, one can determine whether to use an intelligent algorithm for further optimization according to the needs of specific indicators.

(2) Under the same mass ratio μ, no matter the fixed-point theory optimization or the PSO algorithm, it can be found that with increases in the inerter–mass ratio, β, the mean square response decreases, and the lateral spacing between the two peaks becomes larger and larger. That is, the resonant band gradually widens, which shows that the inerter element can reduce the amplitude.

(3) Under the same inerter–mass ratio β, whether using the fixed-point theory or the PSO algorithm, it can be found that when the mass ratio μ increases, the amplitude of the system diminishes, the resonance frequency band expands, and the mean square response value decreases.

**Figure 9:**
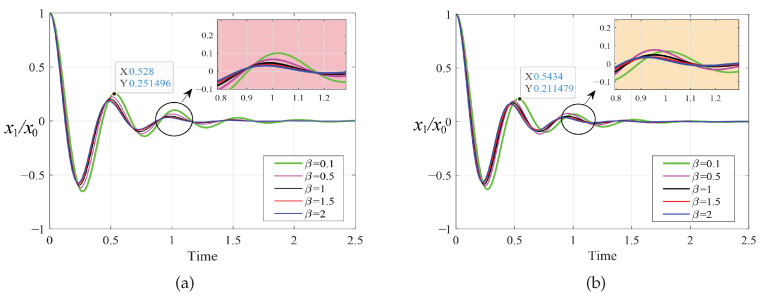
Transient response to initial displacement $x_{0}$ when μ=0.1: (**a**) fixed-point theory optimization and (**b**) particle swarm optimization.

**Figure 10:**
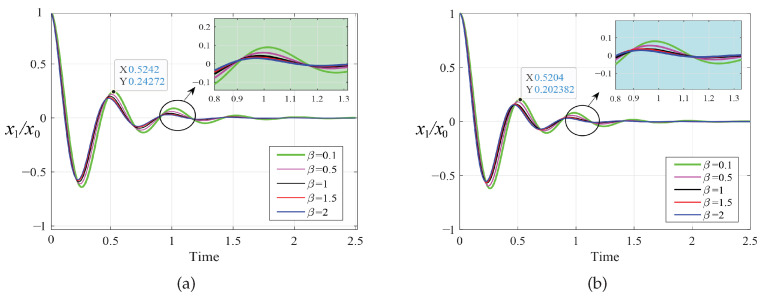
Transient response to initial displacement $x_{0}$ when μ=0.2: (**a**) fixed-point theory optimization and (**b**) particle swarm optimization.

As shown in Figure 9 and Figure 10, we simulated the dimensionless transient response $x_{1}/x_{0}$ of the primary system under the different optimization methods. We selected an initial displacement value of $x_{0}=1\;m$ and a fixed time step of $10^{-4}\;s$. The optimization results of the PSO algorithm are slightly better than the fixed-point theory optimization results. There is no vibration phenomenon when the system is stable in both cases. The corresponding vibration attenuation rate of the primary system when β=2 is obviously faster than in other cases and tends toward a steady state preferentially.

### 3.3. Numerical Simulation

**Figure 11:**
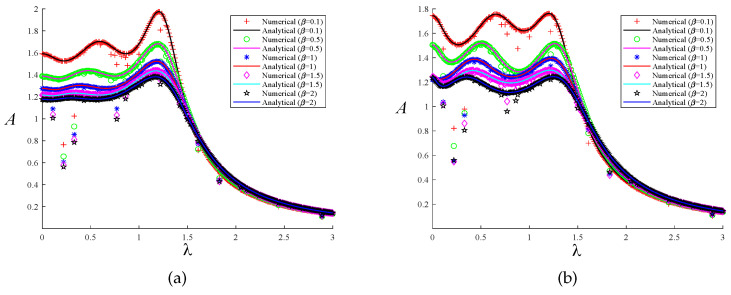
The comparison between the numerical solution and the analytical solution under the two optimization methods when μ=0.1: (**a**) fixed-point theory optimization and (**b**) particle swarm optimization.

**Figure 12:**
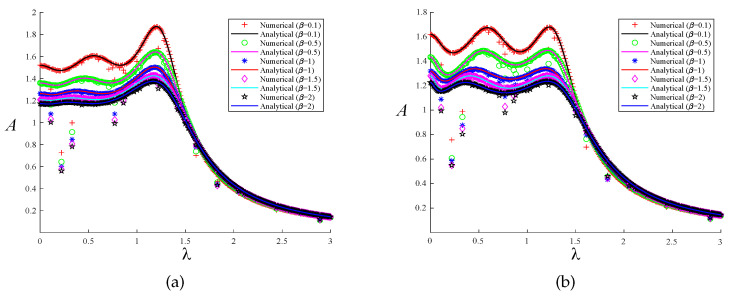
The comparison between the numerical solution and the analytical solution under the two optimization methods when μ=0.2: (**a**) fixed-point theory optimization and (**b**) particle swarm optimization.

A comparison of the analytical and numerical solutions achieved with our two distinct optimization techniques was conducted to authenticate the correctness of the solution process discussed in the previous section. We employed the fourth-order Runge–Kutta technique for numerical simulation, taking 2000s. Assuming that the excitation force amplitude F=1000N, the numerical solutions of the system’s response to the two optimization methods can be obtained. The maximum value of the steady-state solution is taken as the excitation response amplitude, which allows us to obtain the normalized amplitude–frequency curve. When μ=0.1 and μ=0.2, a comparison of the analytical and numerical solutions of the system is presented under the two optimization methods, as shown in Figure 11 and Figure 12. The solid line represents the analytical solution, and the curves drawn by different shapes are the numerical solutions of the system. Different colors indicate different selections of the parameters μ and β. When the system’s parameters are the same, the curves of the two solutions are basically the same regardless of the optimization method, which also verifies the correctness of our theoretical analysis.

## 4. Comparative Analysis of DVA Models under Different Excitation

**Figure 13:**
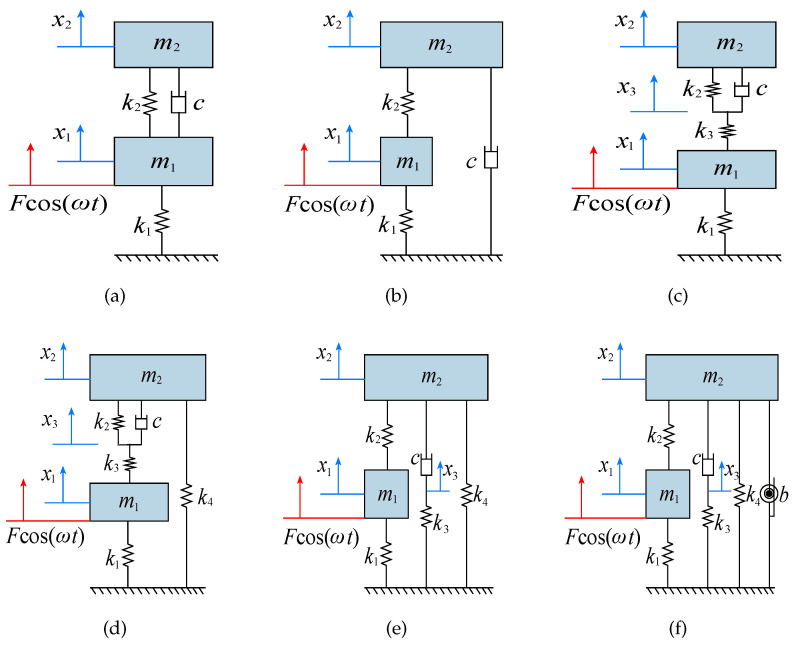
The classical models of DVAs: (**a**) Voigt-type, (**b**) Ren-type, (**c**) TE-type, (**d**) NS-TE-type, (**e**) NS-Shen-type, and (**f**) NS-Wang-type.

In this section, we compare the proposed DVA model with the classical DVA models to prove the effectiveness of the our design. The models involved in the comparison are Voigt-type DVA [2], Ren-type DVA [5], and the DVAs in Ref. [12] (NS-Shen-type DVA), Ref. [13] (NS-Wang-type DVA). Figure 13 displays these DVA models. In addition, comparison is also made between the three-element DVA model without both inerter and negative stiffness (TE-type DVA in Figure 13c), and the three-element DVA model with negative stiffness and without inerter (NS-TE-type DVA in Figure 13d). Setting both $\alpha_{2}=0$ and β=0, the model in Figure 13c is the example of our model. Setting β=0, the model in Figure 13d also is the example of our model. For the convenience of expression, our model is denoted as INS-TE-type DVA.

### 4.1. Harmonic Excitation Scenario

**Figure 14:**
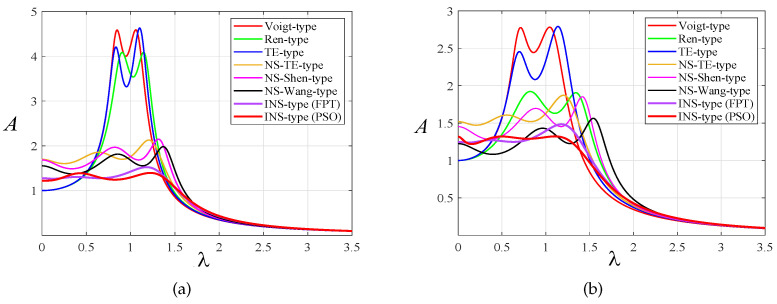
Comparison of amplitude–frequency response curves with other DVAs when β=1: (**a**) μ=0.1 and (**b**) μ=0.3.

According to the optimal parameter expression of each model [2,5,12,13], Figure 14 illustrates the amplitude–frequency response curves of the primary system of each DVA model under harmonic excitation when μ=0.1 and μ=0.3, respectively. When μ=0.3, the optimal parameter values of the proposed model obtained by the PSO algorithm are $\alpha_{1}=1.1941,\alpha_{2}=-0.0898,\nu =0.7360$, and ξ=0.3814. An NS-TE-type DVA model with grounded negative stiffness is superior to the absorption performance achieved by Viogt-type, Ren-type, TE-type DVA, and NS-Shen-type DVAs. The proposed model’s vibration reduction ability is evidently superior to the other six DVA designs when either the fixed-point theory or the PSO algorithm is applied. In addition, we can see that the combination of an inerter and a grounded negative stiffness spring can not only greatly reduce the response amplitude of the primary system but also broaden the frequency band with reduction vibrations. It can be seen from Figure 14 that when the mass ratio μ increases, the lateral distance between the two peaks of the amplitude–frequency curves becomes larger. In summary, the larger the mass ratio μ, the wider the effective vibration reduction frequency band of a DVA and the better the vibration reduction effect.

**Figure 15:**
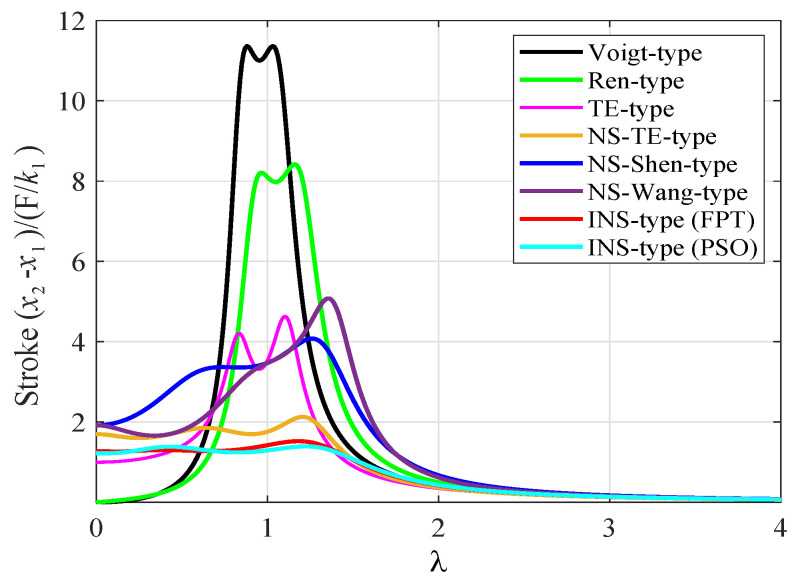
Comparison of frequency response curves of the stroke length with other DVAs when μ=0.1 and β=1.

The stroke length of an absorber is a meaningful index by which to evaluate the performance of a DVA. Figure 15 shows the frequency response of relative movement $x_{2}-x_{1}$ between the primary system and the DVA under a harmonic force, which is also known as stroke length. It can be noted that the proposed three-element-type inerter-based DVA can significantly reduce the peak amplitude of stroke length compared with the classical models, which is conducive to its actual effectuation in more rigorous engineering conditions.

### 4.2. Random Excitation Scenario

There exist many vibration sources in engineering that cannot be described by definite time and space coordinate functions, such as earthquakes, turbulences, uneven road excitation, and noise. These kinds of vibration sources can only be described by probability or statistics. Usually, this type of vibration source is called a random vibration source, and the structural vibration it causes is called a random vibration. Since the mechanical or structural excitation is usually random, it is necessary to prove the effectiveness of our design by comparing its results with those of the classical DVAs under random excitation. The power spectral density function S(ω) of the primary system was taken into account. The symbols V, R, T, NT, NS, NW, and INS stand for a Voigt-type DVA model, Ren-type DVA model, TE-type DVA model, NS-TE-type DVA model, NS-Shen-type DVA model, NS-Wang-type DVA model, and our model, respectively. The primary system displacement mean square values of the seven DVA models can be derived as follows.

$$\begin{array}{c} \begin{array}{cc} \sigma_{V}^{2}= & \int_{-\infty }^{+\infty }S_{V}\left(\omega \right)d\omega =S_{0}\int_{-\infty }^{+\infty }|X_{V_{1}}{|}^{2}d\omega =\frac{\pi S_{0}Y_{V}}{2\mu \xi \omega_{1}^{3}\nu }\\ \sigma_{R}^{2}= & \int_{-\infty }^{+\infty }S_{R}\left(\omega \right)d\omega =S_{0}\int_{-\infty }^{+\infty }|X_{R_{1}}{|}^{2}d\omega =\frac{\pi S_{0}Y_{R}}{2\mu \xi \omega_{1}^{3}\nu^{5}}\\ \sigma_{T}^{2}= & \int_{-\infty }^{+\infty }S_{T}\left(\omega \right)d\omega =S_{0}\int_{-\infty }^{+\infty }|X_{T_{1}}{|}^{2}d\omega =\frac{\pi S_{0}Y_{T}}{2\mu^{4}\xi \omega_{1}^{3}\alpha_{1}^{2}\nu^{2}}\\ \sigma_{NT}^{2}= & \int_{-\infty }^{+\infty }S_{NT}\left(\omega \right)d\omega =S_{0}\int_{-\infty }^{+\infty }|X_{NT_{1}}{|}^{2}d\omega \\ = & \frac{\pi S_{0}Y_{NT}}{2\mu \xi \omega_{1}^{3}\alpha_{1}^{2}\left[\mu \nu^{2}\left(\alpha_{1}\alpha_{2}+\alpha_{1}+\alpha_{2}\right)+\alpha_{1}\alpha_{2}\right]}\\ \sigma_{NS}^{2}= & \int_{-\infty }^{+\infty }S_{NS}\left(\omega \right)d\omega =S_{0}\int_{-\infty }^{+\infty }|X_{NS_{1}}{|}^{2}d\omega \\ = & \frac{\pi S_{0}Y_{NS}}{2\mu \xi \omega_{1}^{3}\nu^{7}\alpha_{1}^{2}\left(1+\alpha_{2}+\mu \alpha_{2}\nu^{2}\right)}\\ \sigma_{NW}^{2}= & \int_{-\infty }^{+\infty }S_{NW}\left(\omega \right)d\omega =S_{0}\int_{-\infty }^{+\infty }|X_{{NW}_{1}}{|}^{2}d\omega \\ = & \frac{\pi S_{0}Y_{NW}}{2\mu \xi \omega_{1}^{3}\nu^{7}\alpha_{1}^{2}\left(\mu \alpha_{2}\nu^{2}+\alpha_{2}+1\right)}\\ \sigma_{INS}^{2}= & \int_{-\infty }^{+\infty }S_{INS}\left(\omega \right)d\omega =S_{0}\int_{-\infty }^{+\infty }|X_{{INS}_{1}}{|}^{2}d\omega \\ = & \frac{\pi S_{0}Y_{INS}}{\xi \omega_{1}^{3}\alpha_{1}^{2}\left[\alpha_{2}\left(\alpha_{2}-2\beta -2\mu \right)+{\left(\mu +\beta \right)}^{2}\right]\left[\mu \nu^{2}\left(\alpha_{1}\alpha_{2}+\alpha_{1}+\alpha_{2}\right)+\alpha_{1}\alpha_{2}\right]} \end{array} \end{array}$$

where

$$\begin{array}{c} \begin{array}{cc} \; & Y_{V}=\mu \nu^{2}\left(2\nu^{2}-1+4\xi^{2}\right)+\mu^{2}\nu^{4}+\nu^{2}\left(\nu^{2}-2+4\xi^{2}\right)+1\\ \; & Y_{R}=\nu^{2}\left(\nu^{2}+4\xi^{2}+\mu -2\right)+1\\ \; & Y_{T}=4\mu \nu^{2}\alpha_{1}\left(\alpha_{1}\mu l_{1}+\mu^{3}\right)\xi^{2}+\mu^{4}\nu^{6}\alpha_{1}l_{2}-\mu^{4}\nu^{4}\alpha_{1}^{2}l_{3}+\mu^{4}\nu^{2}\alpha_{1}^{3}\\ \; & Y_{NT}=4\left(\alpha_{1}\mu n_{1}+\mu n_{2}\right)\left(\mu \nu^{2}n_{3}+\alpha_{1}\alpha_{2}\right)\xi^{2}+\mu^{4}\nu^{6}\left[{\left(1+\mu \right)}^{2}\alpha_{1}^{3}+n_{4}\alpha_{1}^{2}+n_{5}\right]\\ \; & \;\;-\mu^{3}\nu^{4}\alpha_{1}\left(n_{6}\alpha_{1}^{2}-n_{7}\right)+\mu^{2}\nu^{2}\alpha_{1}^{2}n_{8}\left(\mu -\alpha_{2}\right)+\mu \alpha_{2}\alpha_{1}^{3}{\left(\mu -\alpha_{2}\right)}^{2}\\ \; & Y_{NS}=4\xi^{2}\left[1+\alpha_{2}\left(1+\mu \nu^{2}\right)\right]\left\{1+\nu^{2}[\left(\mu^{2}-s_{3}\mu +s_{2}\right)\nu^{2}-2s_{1}\right\}\\ \; & \;\;+\alpha_{1}^{2}\nu^{2}\left\{\left[\mu \left(2\alpha_{2}+1\right)-2{\left(1+\alpha_{2}\right)}^{2}\right]\nu^{2}+s_{4}\nu^{4}+\alpha_{2}+1\right\}\\ \; & Y_{NW}=4\xi^{2}\left(1+\alpha_{2}+\alpha_{2}\mu \nu^{2}\right)\left(\eta_{1}+\eta_{2}\nu^{4}\right)+\alpha_{1}^{2}\nu^{2}\left(\eta_{3}-\eta_{4}\nu^{2}+\eta_{5}\nu^{4}\right)\\ \; & Y_{ISN}=f_{1}\mu^{5}+f_{2}\mu^{4}+f_{3}\mu^{3}+f_{4}\mu^{2}+f_{5}\mu +f_{6} \end{array} \end{array}$$

with

$$\begin{array}{c} \begin{array}{cc} l_{1}= & \left(1+\mu \right)\alpha_{1}-2\mu ,\;l_{2}={\left(1+\mu \right)}^{2}\alpha_{1}^{2}-\mu \left(\mu +2\right),\;l_{3}=\left(\mu +2\right)\alpha_{1}-2\mu \alpha_{1}+\mu^{2}\\ n_{1}= & l_{1},\;n_{2}=\left(\mu -\alpha_{2}\right)\left(\mu +\alpha_{2}\right),\;n_{3}=\alpha_{1}\alpha_{2}+\alpha_{1}+\alpha_{2}\\ n_{4}= & -\mu^{2}+2\left(\alpha_{2}-1\right)\mu +3\alpha_{2},\;n_{5}=\left(\mu -\alpha_{2}\right)\left[\left(\mu -3\alpha_{2}\right)\alpha_{1}+\alpha_{2}\left(\mu -\alpha_{2}\right)\right]\\ n_{6}= & \mu^{2}-2\mu \left(\alpha_{2}-1\right)-3\alpha_{2},\;n_{7}=\left(\mu -\alpha_{2}\right)\left[2\left(\mu -3\alpha_{2}\right)\alpha_{1}+3\alpha_{2}\left(\mu -\alpha_{2}\right)\right]\\ n_{8}= & \left(\mu -3\alpha_{2}\right)\alpha_{1}+3\alpha_{2}\left(\mu -\alpha_{2}\right)\\ s_{1}= & \alpha_{1}+\alpha_{2}-\mu +1,\;s_{2}={\left(\alpha_{1}+\alpha_{2}+1\right)}^{2}\\ s_{3}= & 1+2\left(\alpha_{1}+\alpha_{2}\right),\;s_{4}=\left(1+\alpha_{2}\right)\left[{\left(1+\alpha_{2}\right)}^{2}-2\alpha_{2}\mu \right]+\alpha_{2}\mu^{2}\\ \eta_{1}= & -2\nu^{2}\left(\alpha_{1}+\alpha_{2}+1\right)\left(\beta +1\right)+{\left(1+\beta \right)}^{2}\\ \eta_{2}= & r_{1}+\alpha_{2}\left(2+\alpha_{2}\right)+\alpha_{1}^{2}r_{1}+2\alpha_{1}\left(\alpha_{2}+r_{1}\right)\\ \eta_{3}= & \left(1+\alpha_{2}\right){\left(1+\beta \right)}^{2},\;\eta_{4}=\left(1+\beta \right)\left[2\alpha_{2}\left(2+\alpha_{2}\right)+r_{1}+1\right]\\ \eta_{5}= & 3\alpha_{2}^{2}+\alpha_{2}^{3}+r_{1}^{2}+\alpha_{2}\left(2r_{1}+1\right),\;r_{1}=1+\mu \left(1+\beta \right)\\ f_{1}= & \left(\alpha_{1}^{3}-\alpha_{1}^{2}+\alpha_{1}+\alpha_{2}\right)\nu^{6}\\ f_{2}= & f_{21}\nu^{6}+\left(-\alpha_{1}^{3}+2\alpha_{1}^{2}+3\alpha_{1}\alpha_{2}\right)\nu^{4}\\ f_{21}= & \left(2\beta +2\right)\alpha_{1}^{3}+\left(2\alpha_{2}-2\beta -2\right)\alpha_{1}^{2}+\left(-4\alpha_{2}+2\beta \right)\alpha_{1}-2\alpha_{2}^{2}+2\alpha_{2}\beta \\ f_{3}= & f_{31}\nu^{6}+f_{32}\nu^{4}+f_{33}\nu^{2}\\ f_{31}= & {\left(\beta +1\right)}^{2}\alpha_{1}^{3}+\left(2\alpha_{2}\beta -\beta^{2}+3\alpha_{2}-2\beta \right)\alpha_{1}^{2}+\left(3\alpha_{2}^{2}-4\alpha_{2}\beta +\beta^{2}\right)\alpha_{1}+\alpha_{2}^{3}-2\alpha_{2}^{2}\beta +\alpha_{2}\beta^{2}\\ f_{32}= & \left(2\alpha_{2}-2\beta -2\right)\alpha_{1}^{3}+\left(-8\alpha_{2}+4\beta \right)\alpha_{1}^{2}+\left(-6\alpha_{2}^{2}+6\alpha_{2}\beta \right)\alpha_{1}\\ f_{33}= & \alpha_{1}^{3}+3\alpha_{1}^{2}\alpha_{2}+\left(4\alpha_{2}\xi^{2}+4\xi^{2}\right)\alpha_{1}+4\alpha_{2}\xi^{2} \end{array} \end{array}$$

Supposing that the system parameters of the six DVAs in Figure 13 are selected as μ=0.1 and β=0.1, the corresponding mean square values can be calculated according to their respective optimization formulas [2,5,12,13].

$$\begin{array}{c} \begin{array}{cc} \sigma_{V}^{2} & =\frac{6.401\pi S_{0}}{\omega_{1}^{3}},\;\sigma_{R}^{2}=\frac{5.780\pi S_{0}}{\omega_{1}^{3}}\\ \sigma_{T}^{2} & =\frac{6.098\pi S_{0}}{\omega_{1}^{3}},\;\sigma_{NT}^{2}=\frac{3.095\pi S_{0}}{\omega_{1}^{3}}\\ \sigma_{NS}^{2} & =\frac{3.091\pi S_{0}}{\omega_{1}^{3}},\;\sigma_{NW}^{2}=\frac{3.0474\pi S_{0}}{\omega_{1}^{3}} \end{array} \end{array}$$

We have calculated the mean square responses of the proposed model under the fixed-point theory method of optimization and the PSO algorithm in the previous section. The results show that our models all have lower mean square responses than the above models, which means that our models have better random vibration damping effects than other DVAs, as shown in Figure 13. In addition, NS-TE-type DVAs with only grounded negative stiffness ire superior to Voigt-type DVAs, Ren-type DVAs, and TE-type DVA, while TE-type DVAs without inerters and grounded stiffness are superior to Voigt-type DVAs. Through comparison, this study shows that the simultaneous introduction of an inerter and negative stiffness elements gives the proposed model better vibration absorption performance than other models using the two optimization methods.

**Figure 16:**
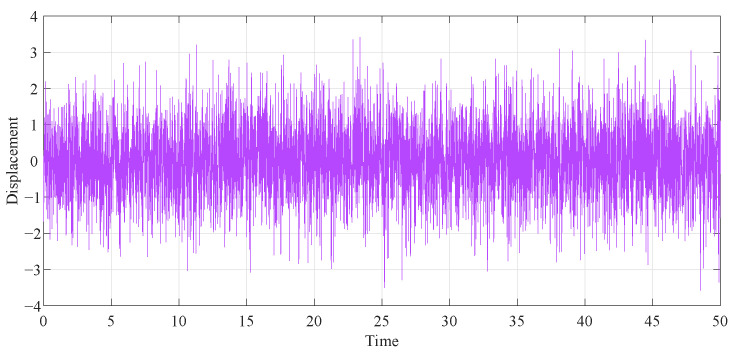
The time history of the random excitation.

**Figure 17:**
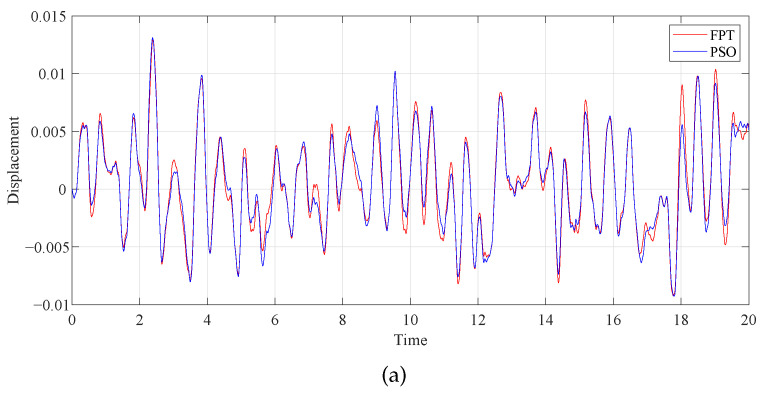

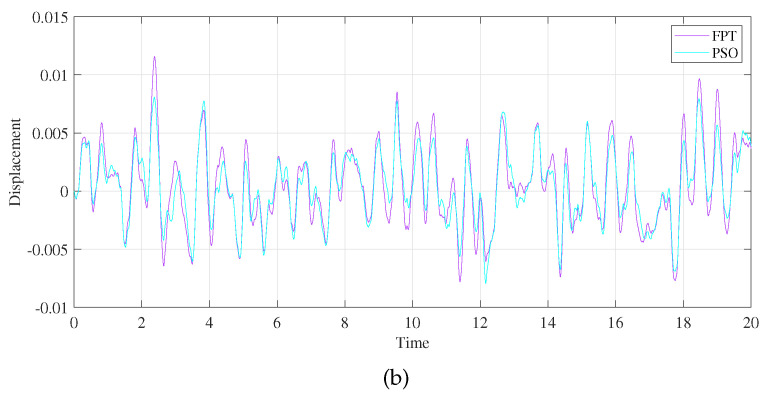
Comparison of the time histories of the primary system with different optimization methods: (**a**) β=0.1 and (**b**) β=1.

**Figure 18:**
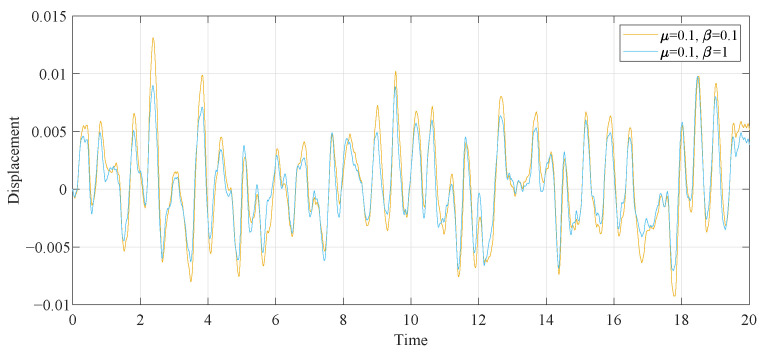
Comparison of the time histories of the primary system with different inerter–mass ratios.

To more accurately illustrate the contrast effect, we employed the fourth-order Runge–Kutta algorithm to generate the time history response diagrams of the primary system attached to various DVAs under 50s of random stimulation, consisting of 5000 normalized random numbers with a zero mean and zero unit (as depicted in Figure 16). First, in the case of the same μ and β parameters, the primary system time history diagrams of the proposed model under two different optimization methods were compared. As illustrated in Figure 17a, when μ=0.1 and β=0.1, the curves of the two optimization methods are roughly the same. Through the specific coordinates of the positioning curve, it can be found that except for individual cases, the displacement of the primary system after intelligent algorithm optimization is smaller than after fixed-point theory optimization. When β is larger, the advantage of the PSO algorithm is more obvious, as shown in Figure 17b. Second, we compared the time history diagrams of the primary system with different values of β, as seen in Figure 18. It was found that increasing the inerter–mass ratios could reduce the response amplitude.

**Figure 19:**
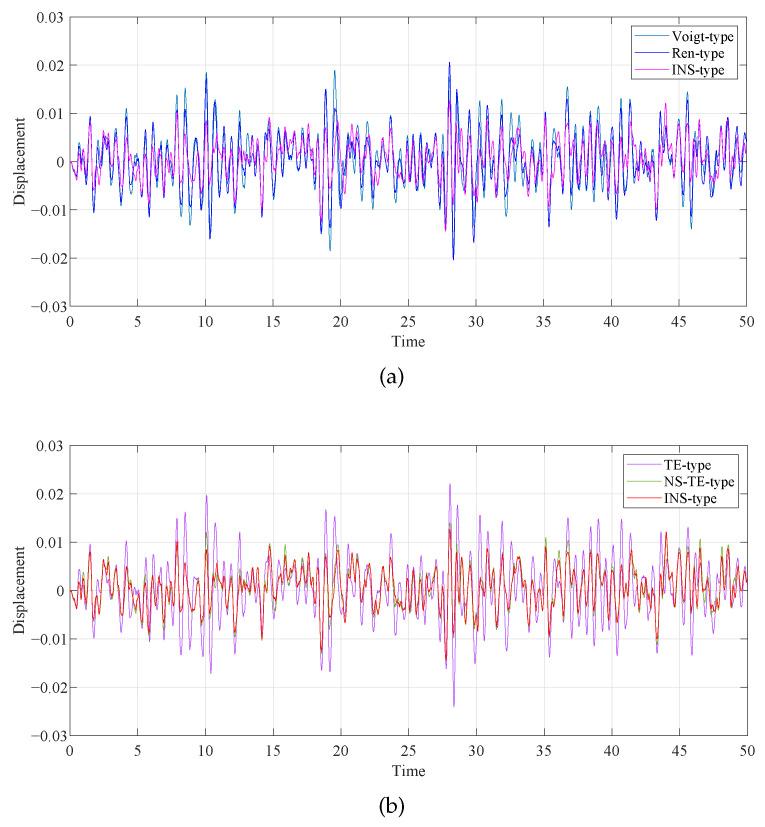

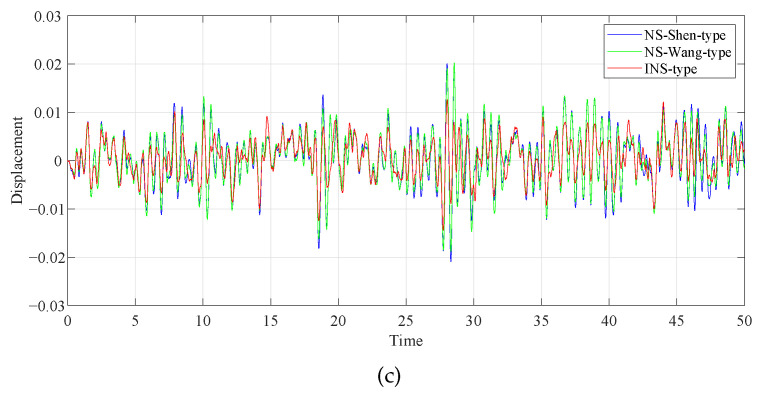
Comparison of the time histories of the primary system with different DVAs: (**a**) Voigt-type and Ren-type; (**b**) TE-type and NS-TE-type; and (**c**) NS-Shen-type and NS-Wang-type.

The primary system time history diagrams of different DVA models when μ=0.1 and β=0.1 are shown in Figure 19. The displacement variance statistics of the primary system and its decreased ratios relative to the uncontrolled primary system are shown in Table 4. After comparing the numerical values, we can find that the variance in the numbers generated by the DVA discussed in this study is smaller than for other models, indicating that the deviation between random variables and mathematical expectations is smaller. The decrease ratios refer to the ratio of the difference between the previous amplitude of the two adjacent waves in the same direction minus the difference between the latter amplitude and the previous amplitude after each fluctuation cycle. The decrease ratios of the wave generated by the primary system without DVA and the wave generated by the primary system with DVA were calculated. It was found that the decrease ratio of the model in this paper is larger. The proposed model exhibits superior performance compared to other DVAs under random excitation. Furthermore, compared to the TE-type DVA and NS-TE-type DVA models, the NS-TE-type DVA model displays superior control performance, indicating that the introduction of grounded negative stiffness to the DVA model has a beneficial random vibration reduction effect. The combined application of the inerter and negative stiffness has a satisfactory vibration reduction effect and can provide ideas and beneficial choices for the design of vibration isolation systems in seismic engineering.

## 5. Conclusions and Prospect

The vibration experienced under complex working conditions is an important factor affecting the efficiency, reliability, accuracy, and safety of engineered structures. It is a technical problem and a frontier in the field of major engineering to suppress harmful vibrations in complex dynamic environments. In our study, a novel three-element-type inerter-based DVA model with grounded negative stiffness is presented. The specific expressions and optimal values of the frequency ratio, $\alpha_{1}$, $\alpha_{2}$, ν, and ξ, are obtained by the fixed-point theory and provide an iterative value range for algorithm optimization. In order to further minimize the maximum amplitude amplification coefficient of the primary system, an intelligent PSO algorithm is introduced to optimize the four parameters iteratively at the same time. By tracking the individual and global optimal values, the particle alters its velocity and location, and the ultimate output parameters bring the optimized curve to nearly equal heights. The specific values of the response characteristics of the primary system under the fixed-point theory optimization method and the intelligent PSO algorithm are compared more comprehensively. It is found that after using the PSO algorithm, the two resonance peaks on the amplitude–frequency response curve are almost equal, the corresponding amplitude–frequency response is significantly reduced, the mean square value is lower, and the response curve is more stable. The numerical and analytical solutions are simulated to further prove the validity of the optimal parameter values.

Finally, from the perspective of amplitude–frequency curves, stroke lengths, time history diagrams, displacement variances, and decrease ratios, the control performance of the proposed model under harmonic excitation and random excitation is compared with other DVAs. The results verify that the proposed model has significant advantages in reducing the harmonic and random vibration, and the optimization effect of the intelligent algorithm is better. The results provide theoretical and algorithmic support for the structural design and parameter optimization of DVAs. Beam structures are a common structural form in engineering applications. In addition to studying the vibration reduction performance of a single vibration absorber, multiple or distributed vibration absorbers are also a research frontier in the vibration suppression of beam structures. In our future research, we will combine the actual needs of bridges regarding seismic resistance and buildings regarding seismic protection, apply the model proposed in this paper to distributed DVAs, and combine traditional theory and intelligent algorithms to further improve the structural parameters of DVAs.

## Figures and Tables

**Table 1:** The optimal parameter values of the system under different conditions based on fixed-point theory.

**Case 1:**	μ= 0.1			
β	$\alpha_{1}$	$\alpha_{2}$	ν	ξ
0.1	0.3612	−0.1078	1.6179	0.2342
0.5	0.7116	−0.1061	1.5007	0.3072
1.0	0.9775	−0.0909	1.3659	0.3541
1.5	1.1516	−0.0780	1.2578	0.3822
2.0	1.2749	−0.0679	1.1706	0.4011
**Case 2:**	μ= 0.2			
β	$\alpha_{1}$	$\alpha_{2}$	ν	ξ
0.1	0.4680	−0.1110	1.1249	0.2583
0.5	0.7756	−0.1031	1.0405	0.3191
1.0	1.0179	−0.0880	0.9491	0.3608
1.5	1.1795	−0.0757	0.8760	0.3866
2.0	1.2954	−0.0661	0.8169	0.4042

**Table 2:** The optimal parameter values of the system under different conditions by the PSO algorithm.

**Case 1:**	μ= 0.1			
β	$\alpha_{1}$	$\alpha_{2}$	ν	ξ
0.1	0.38226	−0.11283	1.60040	0.24288
0.5	0.71506	−0.11568	1.53110	0.36068
1.0	0.99482	−0.08441	1.37350	0.39000
1.5	1.18000	−0.07805	1.20740	0.42300
2.0	1.28910	−0.07451	1.16210	0.46160
**Case 2:**	μ= 0.2			
β	$\alpha_{1}$	$\alpha_{2}$	ν	ξ
0.1	0.48487	−0.11701	1.13620	0.28304
0.5	0.78925	−0.10323	0.98870	0.34579
1.0	1.08310	−0.09290	0.93500	0.39130
1.5	1.20780	−0.07956	0.83454	0.43079
2.0	1.34370	−0.07102	0.79453	0.44900

**Table 3:** Numerical comparison of the response characteristics of the primary system under two different optimization methods.

**Case 1:**	μ= 0.1				
β	$A_{max}$	$\lambda_{peak1}$	$\lambda_{peak2}$	$|\lambda_{peak2}-\lambda_{peak1}|$	$\sigma_{opt}^{2}\left(\frac{\pi S_{0}}{\omega_{1}^{3}}\right)$
β = 0.1 (FPT)	1.97454	0.59	1.21	0.62	2.7820
β = 0.1 (PSO)	1.76320	0.65	1.20	0.55	2.7111
β = 0.5 (FPT)	1.68205	0.47	1.19	0.72	2.2072
β = 0.5 (PSO)	1.51950	0.50	1.26	0.76	2.1109
β = 1 (FPT)	1.52233	0.37	1.18	0.81	1.9117
β = 1 (PSO)	1.39426	0.43	1.23	0.80	1.8351
β = 1.5 (FPT)	1.43656	0.32	1.18	0.86	1.7570
β = 1.5 (PSO)	1.29750	0.42	1.22	0.80	1.6714
β = 2 (FPT)	1.38211	0.29	1.18	0.89	1.6612
β = 2 (PSO)	1.24456	0.35	1.26	0.91	1.5603
**Case 2:**	μ= 0.2				
β	$A_{max}$	$\lambda_{peak1}$	$\lambda_{peak2}$	$|\lambda_{peak2}-\lambda_{peak1}|$	$\sigma_{opt}^{2}\left(\frac{\pi S_{0}}{\omega_{1}^{3}}\right)$
β = 0.1 (FPT)	1.87156	0.55	1.20	0.65	2.5747
β = 0.1 (PSO)	1.67845	0.59	1.24	0.65	2.4914
β = 0.5 (FPT)	1.68166	0.46	1.20	0.74	2.1280
β = 0.5 (PSO)	1.48809	0.54	1.22	0.68	2.0644
β = 1 (FPT)	1.52295	0.38	1.18	0.72	1.8735
β = 1 (PSO)	1.33904	0.44	1.20	0.76	1.7753
β = 1.5 (FPT)	1.43656	0.33	1.18	0.85	1.7344
β = 1.5 (PSO)	1.28069	0.40	1.24	0.84	1.6450
β = 2 (FPT)	1.38211	0.27	1.11	0.84	1.6463
β = 2 (PSO)	1.23473	0.35	1.23	0.88	1.5500

**Table 4:** The variances and decrease ratios of primary structure displacement.

Models	Variances	Decrease Ratios (%)
Without DVA	2.5712 × 10${}^{-4}$	/
Voigt-type DVA	3.5639 × 10${}^{-5}$	86.14
Ren-type DVA	3.0313 × 10${}^{-5}$	88.21
TE-type DVA	4.0445 × 10${}^{-5}$	84.27
NS-TE-type DVA	1.8508 × 10${}^{-5}$	92.80
NS-Shen-type DVA	2.9562 × 10${}^{-5}$	88.50
NS-Wang-type DVA	2.8035 × 10${}^{-5}$	89.10
INS-type DVA (β=0.1)	1.6080 × 10${}^{-5}$	93.75
INS-type DVA (β=0.5)	1.1951 × 10${}^{-5}$	95.35
INS-type DVA (β=1)	1.0637 × 10${}^{-5}$	95.86
INS-type DVA (β=1.5)	9.8359 × 10${}^{-6}$	96.17
INS-type DVA (β=2)	9.3692 × 10${}^{-6}$	96.36

## Data Availability

The data presented in this study are available on request from the corresponding author.
